# Fast Determination of Eleven Food Additives in River Water Using C18 Functionalized Magnetic Organic Polymer Nanocomposite Followed by High-Performance Liquid Chromatography

**DOI:** 10.3390/molecules29153675

**Published:** 2024-08-02

**Authors:** Chao Lei, Shun Zhang, Wen-Xin Liu, Ming-Li Ye, Yong-Gang Zhao

**Affiliations:** 1 College of Biological and Environmental Engineering, Zhejiang Shuren University, Hangzhou 310015, China; chaolei_212@163.com (C.L.); lwx187221@163.com (W.-X.L.); 2 Guoke Ningbo Life Science and Health Industry Research Institute, Ningbo 315010, China; zhangshun@ucas.ac.cn; 3 College of Environment, Zhejiang University of Technology, Hangzhou 310014, China

**Keywords:** magnetic dispersive solid phase extraction, food additives, high-performance liquid chromatography, magnetic nanomaterial

## Abstract

A novel magnetic nanomaterial with Fe_3_O_4_ as the core, PS-DVB as the shell layer, and the surface modified with C18 (C18−PS−DVB−Fe_3_O_4_) had been synthesized by seeded emulsion polymerization. C18−PS−DVB−Fe_3_O_4_ retains the advantages of the chemical stability, large porosity, and uniform morphology of organic polymers and has the magnetic properties of Fe_3_O_4_. A simple, flexible, and efficient magnetic dispersive solid phase extraction (Mag-dSPE) method for the extraction of preservatives, sweeteners, and colorants in river water was established. C18−PS−DVB−Fe_3_O_4_ was used as an adsorbent for Mag-dSPE and was coupled with high-performance liquid chromatography (HPLC) to detect 11 food additives: acesulfame, amaranth, benzoic acid, tartrazine, saccharin sodium, sorbic acid, dehydroacetic acid, sunset yellow, allura red, brilliant blue, and erythrosine. Under the optimum extraction conditions, combined with ChromCoreTMAQC18 (5 μm, 4.6 × 250 mm), 20 mmol/L ammonium acetate aqueous solution and methanol were used as mobile phases, and the detection wavelengths were 240 nm and 410 nm. The limits of detection (LODs) of 11 food additives were 0.6–3.1 μg/L with satisfactory recoveries ranging from 86.53% to 106.32%. And the material could be reused for five cycles without much sacrifice of extraction efficiency. The proposed method has been used to determine food additives in river water samples, and results demonstrate the applicability of the proposed C18−PS−DVB−Fe_3_O_4_ Mag-dSPE coupled with the HPLC method to environment monitoring analysis.

## 1. Introduction

With the increasing pursuit of an improved quality of life, people have much higher requirements for the quality of food. In order to improve the appearance, flavor, taste, and storage of food, food additives are usually used in the food industry [1]. These food additives, according to their function, can be divided into colorants, preservatives, sweeteners, thickeners, emulsifiers, enhancers, and acidity regulators, etc. [2,3,4]. The rational use of these food additives not only improves the color and taste of food, but also improves the nutritional value. However, the improper use of some food additives has led to potential threats to human health. In addition, some food additives have been shown to be potential causes of disease [5,6,7]. For example, Lakshmi and co-workers found that isatins may cause permanent damage to the cornea and conjunctiva [8]. Ma and co-workers reported that excessive intake of isatin has mutagenic effects and may lead to tumors. In addition, some highly toxic synthetic chemicals are also used as illegal food additives, causing serious food safety accidents [9]. In addition, in the production, processing, packaging, transportation, and storage of food additives, food additives will flow into the environment due to improper operation [10]. Benzoic acid, which is often used for food preservation, was detected in groundwater at concentrations of 10~27,500 μg/L [11]. Acesulfame is used to improve the taste of food, and the concentration detected in surface water can be as high as 21 μg/L [12]. In addition, allura red, which is used for food coloring, was detected in natural water samples at concentrations ranging from 9.9 to 77.5 μg/L [13]. Therefore, the adsorption removal and enrichment detection of food additives in environmental water samples is an urgent problem to be solved.

In the production, processing, packaging, transportation, and storage of food additives, food additives will flow into the environment because of improper operation. In addition, many synthetic food additives are difficult to degrade in the body, and they are released into the environment through excretion. The ultimate destination of these food additives would be transferred to the surface water. And the decomposition of a variety of aromatic amines display carcinogenicity. Therefore, it is worth studying to establish a simple and feasible method for the detection of food additives in environmental water samples [14,15,16,17].

At present, the detection methods of food additives include liquid chromatography [18,19,20,21,22], gas chromatography [23], and colorimetric methods [24]. However, the composition of the sample is very complex, and the sample needs to be pretreated before the instrument detection. Solid phase extraction (SPE) is the most widely used pretreatment technique. It includes solid phase microextraction (SPME), dispersed-solid phase extraction (d-SPE) [25], and molecularly imprinted solid phase extraction (MISPE) [26,27,28]. SPE minimizes matrix effects, improves selectivity and recovery, and the detection limit of the instrument is greatly reduced [29]. In 2022, a novel molecularly imprinted polymer was used as an adsorbent for MISPE to remove sodium saccharin from the sample. The detection limit was 7.0 g/L [30]. SPE is more efficient and flexible than liquid–liquid extraction in terms of flexibility, simplicity, and low consumption of organic solvents. Still, the solid–liquid separation time is long after the target is adsorbed by the adsorbent. Therefore, an obvious trend in analytical chemistry is to shorten the separation time. To solve this problem, a simple and efficient magnetic dispersive solid phase extraction (Mag-dSPE) technique is developed. Mag-dSPE has the advantages of rapid solid–liquid separation, a simple method, and low cost, and it is suitable for complex matrices. The use of Mag-dSPE greatly reduces the consumption of organic solvents, which is an environmentally friendly technology [31,32,33,34]. So, Mag-dSPE is also widely used in the pretreatment of food additives in recent years [35,36,37,38,39]. In 2020, a novel magnetic porous nanofiber was used as an adsorbent for Mag-dSPE and HPLC to detect Sudan dye in food, with a detection limit of 0.88–1.27 μg/L [40].

In this study, in order to improve the preferential adsorption ability of magnetic nanomaterials to weak polar or non-polar compounds, the materials were modified by C18. C18 modification is a commonly used functional method in reversed-phase chromatography. The working principle of reversed-phase chromatographic packing is to separate the components of a chemical mixture at an appropriate flow rate by making use of the difference in affinity between chemical substances with different chemical phases and fillers. Its separation is based on the difference between the hydrophilicity and hydrophobicity of compounds. Therefore, after the modification of C18, the specific adsorption ability of the material to non-polar compounds was increased.

**Figure 1:**
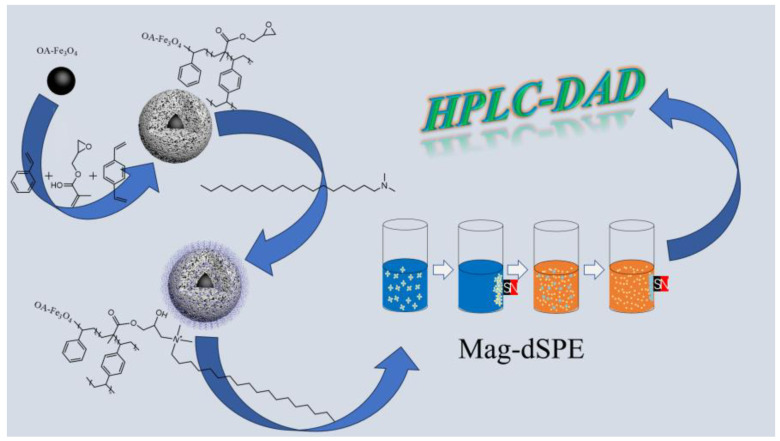
Preparation of C18−PS−DVB−Fe_3_O_4_ and the Mag-dSPE process.

At present, C18 materials are mainly silicon-based materials. Compared with organic resin, silicon-based materials have no advantage in preparation or cost. In order to prepare a kind of material which can replace silicon-based C18 material, a novel magnetic nanomaterial with Fe_3_O_4_ as the core, PS-DVB as the shell layer, and the surface modified with C18 (C18−PS−DVB−Fe_3_O_4_) was synthesized. C18−PS−DVB−Fe_3_O_4_ is used as a powerful sorbent in Mag-dSPE for the separation and preconcentration of 11 food additives. In addition, an analytical procedure combining the Mag-dSPE technique with HPLC has been proposed (Figure 1). The excellent sensitivity of the developed method for 11 food additives was investigated in laboratory batch tests, and it can be applied to the routine analyses for the determination of food additives in environmental water samples.

## 2. Results and Discussion

### 2.1. Characterization of C18−PS−DVB−Fe_3_O_4_

In this study, a novel nanomaterial of C18−PS−DVB−Fe_3_O_4_ was synthesized, and the excellent properties of C18−PS−DVB−Fe_3_O_4_ were characterized by SEM, VSM, XPS, and BET.

#### 2.1.1. Morphological Characteristics

**Figure 2:**
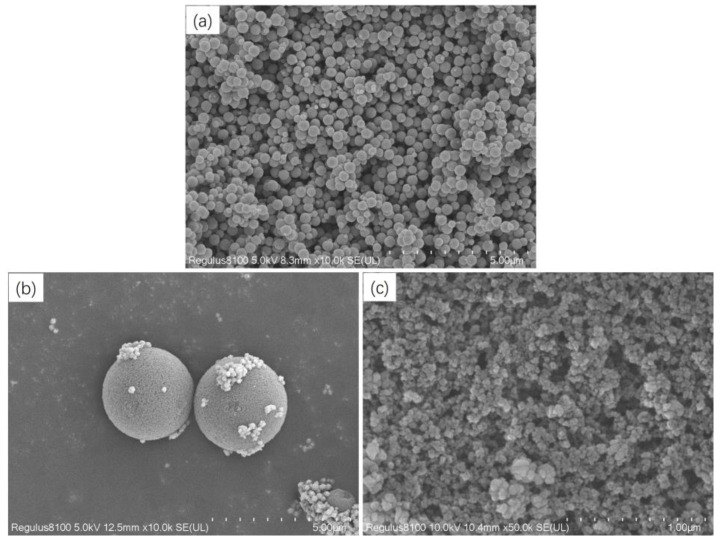
SEM images of nano Fe_3_O_4_ (**a**), C18−PS−DVB−Fe_3_O_4_ (**b**), and a high-definition picture of C18−PS−DVB−Fe_3_O_4_ (**c**).

**Figure 3:**
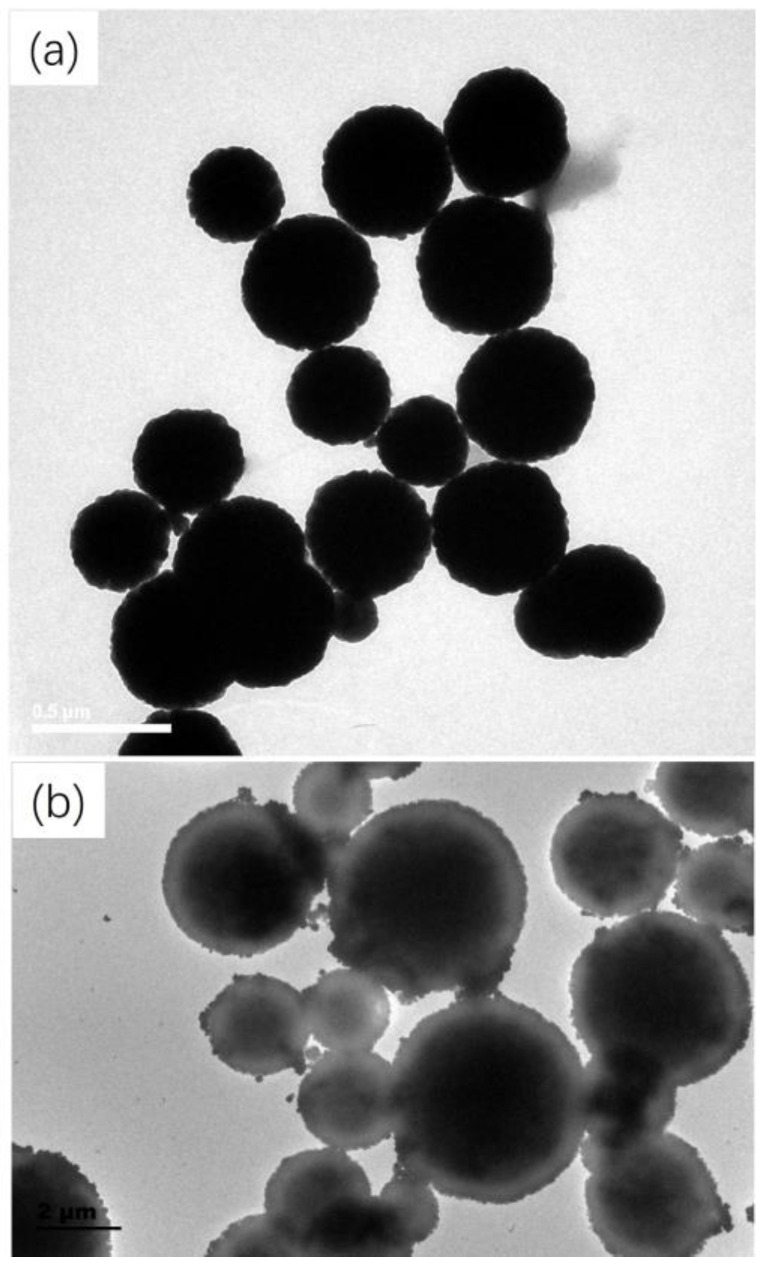
TEM images of Fe_3_O_4_ (**a**) and C18−PS−DVB−Fe_3_O_4_ (**b**).

The morphological features of Fe_3_O_4_ and C18−PS−DVB−Fe_3_O_4_ were investigated by SEM and TEM. As shown in Figure 2a, the SEM image exhibited that the particle size of Fe_3_O_4_ was uniform. The C18−PS−DVB−Fe_3_O_4_ nanoparticles had a good spherical shape, a smooth surface, and many pores. Combined with the Figure 2b,c and Figure 3, the uniformity of the particle size needs to be improved. However, C18−PS−DVB−Fe_3_O_4_ presented an image of the core-shell structure. This was because the electron beam was difficult to pass through Fe_3_O_4_, but it can pass through organic resin.

#### 2.1.2. Magnetic Property

**Figure 4:**
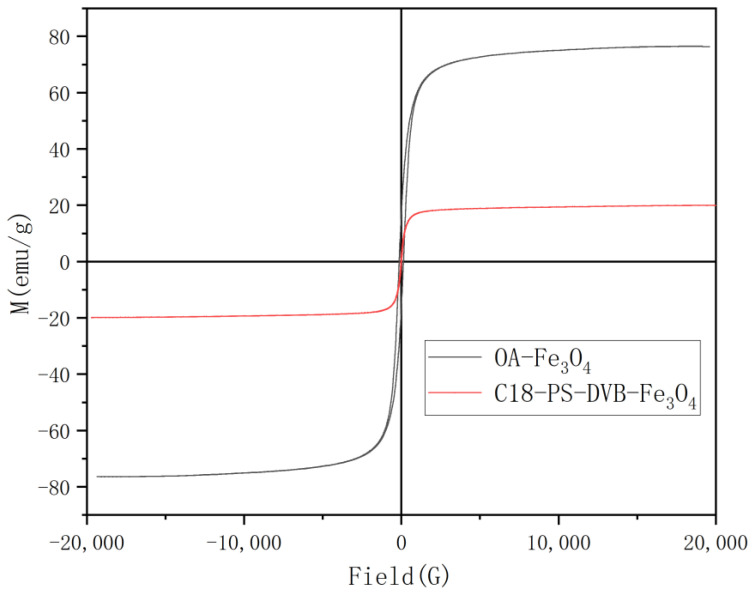
Magnetization curves of Fe_3_O_4_ and C18−PS−DVB−Fe_3_O_4_ nanoparticles.

Figure 4 showed the magnetic performance of Fe_3_O_4_ and C18−PS−DVB−Fe_3_O_4_, which were analyzed by VSM. The magnetic hysteresis loops reflect that the saturation value of Fe_3_O_4_ was 62.67 emu/g, and C18−PS−DVB−Fe_3_O_4_ was 19.93 emu/g. The saturation magnetization of C18−PS−DVB−Fe_3_O_4_ was obviously lower than that of Fe_3_O_4_, which was related to the organic polymer layer that coated the Fe_3_O_4_ surface effectively. However, it had no remarkable influence on the rapid magnetic separation of the adsorbent from the sample solution. C18−PS−DVB−Fe_3_O_4_ can not only be evenly dispersed in a solution without an external magnetic field, but can also be rapidly separated from the solution via a magnet within 10 s.

#### 2.1.3. XPS Analysis

**Figure 5:**
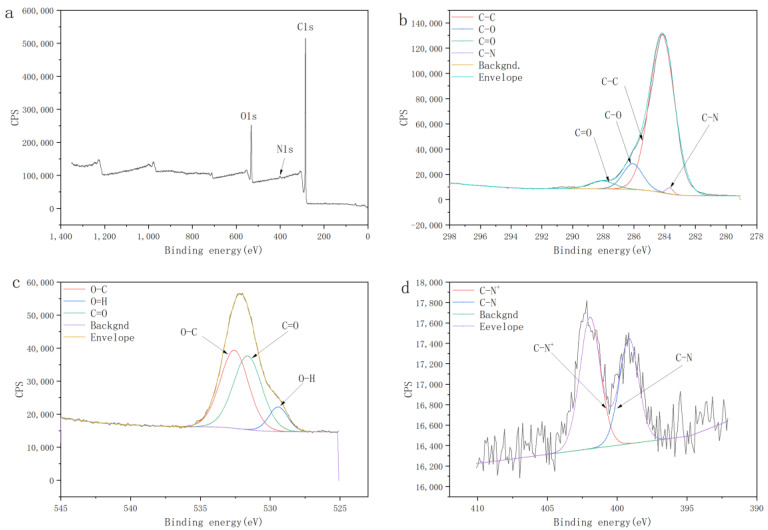
X-ray photoelectron spectroscopy (XPS) analysis (**a**), the fit of the C1s XPS spectrum (**b**), the fit of the O1s XPS spectrum (**c**), the fit of the N1s XPS spectrum (**d**).

The chemical composition of C18−PS−DVB−Fe_3_O_4_ was investigated by XPS. As shown in Figure 5a, the wide scan XPS spectra showed photoelectron lines at a binding energy of about 284.18 eV and 532.12 eV, attributed to carbon (C1s) and oxygen (O1s). The C1s high-resolution scan could be fitted into four peaks (Figure 5b) with binding energies of 283.68 eV, 284.18 eV, 286.08 eV, and 288.08 eV, which were attributed to C-N, C-H, C-O, and O-C=O, respectively. The O1s high-resolution scan could be fitted into three peaks (Figure 5c) with binding energies of 529.42 eV, 531.62 eV, and 532.57 eV, which were attributed to O-H, O=C, and C-O, respectively. The successful functionalization was affirmed by the appearance of an N1s signal between 395 eV and 405 eV in a high-resolution XPS spectrum (Figure 5d). The N1s high-resolution scan could be fitted into two peaks with binding energies of 401.94 eV and 399.31 eV, which were attributed to C-N and C-N^+^, respectively. And no characteristic signal for iron (Fe 2p) between 710 eV and 740 eV in the high-resolution XPS spectrum was observed, confirming that there was no leakage of Fe_3_O_4_ on the surface of C18−PS−DVB−Fe_3_O_4_.

#### 2.1.4. Specific Surface Area Analysis

To further investigate the entirely porous natures of C18−PS−DVB−Fe_3_O_4_, N_2_ adsorption–desorption measurements were performed (as shown in Figure S1a). The N_2_ adsorption isotherm accords with the type III isotherm. The test results showed that the specific surface area of this material was 372.00 m^2^/g. From the pore size distribution calculated by the desorption branch of the isotherm of the particles by the BJH method (Figure S1b), the pore size range of the material was between 0 nm and 70 nm, and the mesopore of 3.0–4.0 nm was dominant, which showed that the material has a good pore structure (including micropores, mesopores, and macropores).

In addition, it was compared with the materials without C18 modification (Figure S2). The specific surface area of PS−DVB−Fe_3_O_4_ was 415.25 m^2^/g, which was lower than that of C18 modified, which may be due to the blocking of some voids after C18 grafting.

#### 2.1.5. FTIR Analysis

**Figure 6:**
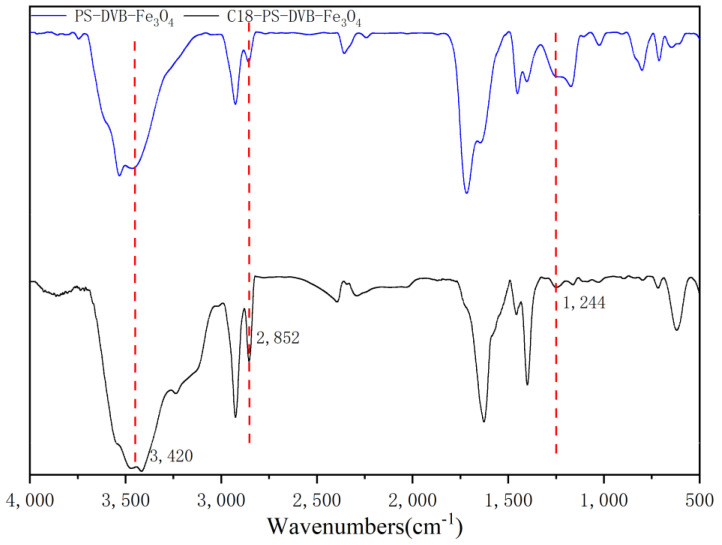
FTIR analysis of PS−DVB−Fe_3_O_4_ and C18−PS−DVB−Fe_3_O_4_.

In order to further identify the functional groups on the surface of the synthesized sample, FTIR test was used, as shown in Figure 6. The tensile vibration peak at 3420 cm^−1^ corresponds to the -OH functional group. The absorption peak at 2852 cm^−1^ belongs to methylene, and the peak intensity of PS−DVB−Fe_3_O_4_ at this position is significantly weaker than that of C18−PS−DVB−Fe_3_O_4_, which was mainly due to the rich methylene ((-CH_2_)_17_) in N, N-dimethyloctadecylamine. The absorption peak at 1244 cm^−1^ was the epoxy group, and the peak intensity of PS−DVB−Fe_3_O_4_ at this position is significantly stronger than that of C18−PS−DVB−Fe_3_O_4_ because the C18 functional group was grafted by the epoxy ring opening reaction. According to the increase in the intensity of the methylene peak and the decrease in the intensity of the epoxy group peak, it was shown that the C18 functional group was successfully grafted.

#### 2.1.6. Nitrogen Content Analysis

The Elementar Vario EL element analyzer was used to analyze the nitrogen content of the sample, and the results are shown in Table 1. In the synthesis process of C18 functionalized nano Fe_3_O_4_ magnetic polymer materials, GMA is the only oxygen source, and N, N-dimethyloctadecylamine was the only nitrogen source. Therefore, the grafting rate of C18 functional groups can be calculated by detecting the oxygen and nitrogen content in the materials. It could be concluded from this Table that the content of O atoms in the non-functionalized nano-magnetic polymer composite was 9.6%, that the content of GMA in the material was 0.40 g/g, and that there was 2.546 mmol of the epoxy group in the 1.0 g magnetic polymer composite. After C18 functionalization, O content decreased to 8.5%, while N content was 1.5%. It can be calculated that the contents of GMA and N, N-dimethyloctadecylamine in the 1.0 g C18 functionalized magnetic polymer composite were 0.25 g/g and 0.30 g/g, respectively. In the 1.0 g magnetic polymer composite, the C18 functional group exists at 1.766 mmol and the epoxy group exists at 1.035 mmol. It can be concluded that the grafting rate of the C18 functional group was 58.6%.

### 2.2. C18−PS−DVB−Fe_3_O_4_ Mag-dSPE Procedure and Its Optimization

In this work, C18−PS−DVB−Fe_3_O_4_ nanocomposites were used as adsorbents for 11 food additives, and then the target substances were extracted from environmental water samples by the Mag-dSPE procedure. In this process, the type and concentration of the eluent, amount of adsorbent, pH of the water sample, and volume of the water sample will affect the recovery of the analyte.

#### 2.2.1. Optimization of Elution Solvent

**Figure 7:**
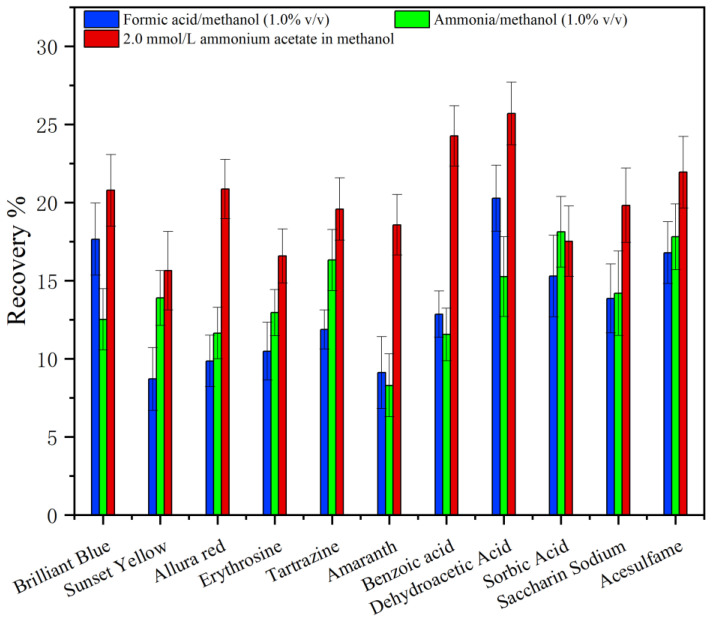
Effect of the type of desorption solvent on the recovery (*n* = 6).

**Figure 8:**
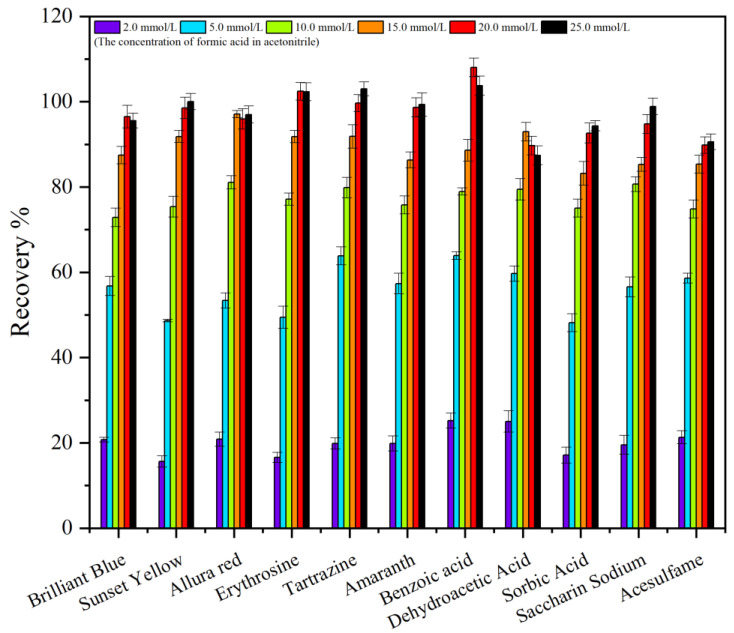
Influence of different concentrations of the ammonium acetate–methanol solution on the recovery (*n* = 6).

The selection of a suitable elution solvent was an important aspect of improving the extraction efficiency. The ideal elution solvent can completely elute the analyte from the surface of the extraction material. In this study, several solutions of formic acid/methanol (1.0%, *v*/*v*), ammonia/methanol (1.0%, *v*/*v*), 2.0 mmol/L ammonium acetate in methanol, 5.0 mmol/L ammonium acetate in methanol, 10.0 mmol/L ammonium acetate in methanol, 15.0 mmol/L ammonium acetate in methanol, 20.0 mmol/L ammonium acetate in methanol, and 25.0 mmol/L ammonium acetate in methanol were studied. The results are shown in Figure 7 and Figure 8. The elution effect of the 2.0 mmol/L ammonium acetate–methanol solution was obviously better than formic acid/methanol (1.0%, *v*/*v*) and ammonia/methanol (1.0%, *v*/*v*). Using 2.0 mmol/L ammonium acetate in methanol, the recoveries of 11 food additives were 15.62–24.3%. The possible reason was that the mixture of ammonium acetate and methanol destroys the electrostatic force between C18−PS−DVB−Fe_3_O_4_ and analytes. Compared with the ammonium acetate solution of 2.0 mmol/L, using the ammonium acetate solution of 20.0 mmol/L as the eluent, the elution effect of 11 kinds of food additives was the best, and the recoveries were 89.8–108.0%. It may be explained that the higher the concentration of ammonium acetate, the greater the destruction intensity of the electrostatic force between C18−PS−DVB−Fe_3_O_4_ and the analyte.

#### 2.2.2. Optimization of Adsorbent Dosage

**Figure 9:**
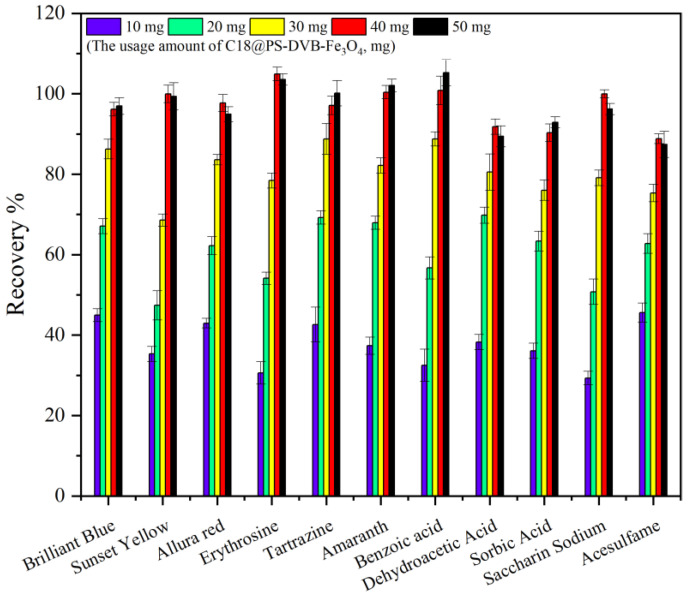
Effect of adsorbent dosage on the recovery (*n* = 6).

The control of the adsorption dose has a great influence on the adsorption effect of the target substance. If the adsorption dose is too small, the target can not be completely adsorbed, and if the adsorption dose is too much, there will be too many irreversible points on the surface of the adsorbent so that the adsorbed target can not be eluted successfully, thus affecting the elution efficiency. In this paper, adsorbent dosages such as 10 mg, 20 mg, 30 mg, 40 mg, and 50 mg are selected for research. According to the experiment, the recoveries of 11 kinds of food additives were obtained. As shown in Figure 9, the trend of recovery was obvious when the amount of adsorbent was in the range of 10–50 mg. The adsorption effect was best when the amount of adsorbent was 40 mg, and the recoveries of 11 food additives were 88.2–105.2%. When the amount of adsorbent increased to 50 mg, the recovery did not change significantly. Therefore, the adsorbent of 40 mg is the best dosage.

#### 2.2.3. Optimization pH of Water Sample

**Figure 10:**
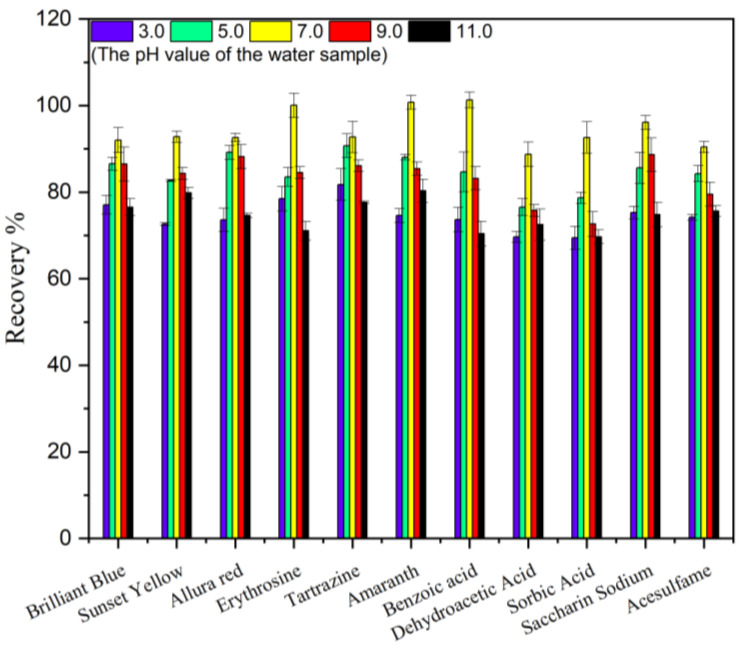
Effect of pH on the recovery (*n* = 6).

Various pH values from 3.0 to 11.0 were used to optimize the Mag-dSPE procedure, and the results were shown in Figure 10. It indicated that, with the increasing of pH values from 3.0 to 11.0, there was no significant effect on the recovery of the target analytes, and the recoveries were in the range of 70.1–101%. With the increasing of pH values from 5.0 to 7.0, the satisfactory recoveries of the 11 food additives increased slightly. Above pH 7.0, the recoveries of 11 food additives decreased with the increase in pH within the studied pH range. Above all, 7.0 was chosen as the pH value for the investigations that followed.

#### 2.2.4. Optimization of Volume of Water Sample

**Figure 11:**
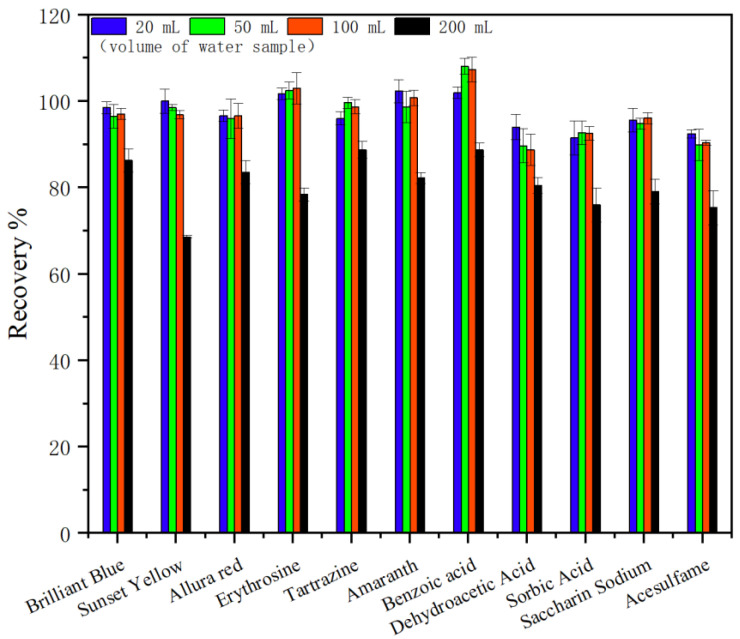
Effect of volume on the recovery (*n* = 6).

To obtain reliable analytical results and a high enrichment factor, obtaining satisfactory recoveries of the target substance from a large volume of sample solutions is very important. Maximum volumes ranging from 20 mL to 200 mL were studied. Following the experimental procedure, the recoveries of 11 food additives at different volumes were obtained, and the results indicate that the maximum volume was as high as 100 mL with a recovery >88.7% (Figure 11). Therefore, 100 mL of the sample solution was adopted for the preconcentration of 11 food additives from sample solutions. A high enrichment factor of 100 times was obtained because the final elute solution was 1.0 mL in these experiments.

**Figure 12:**
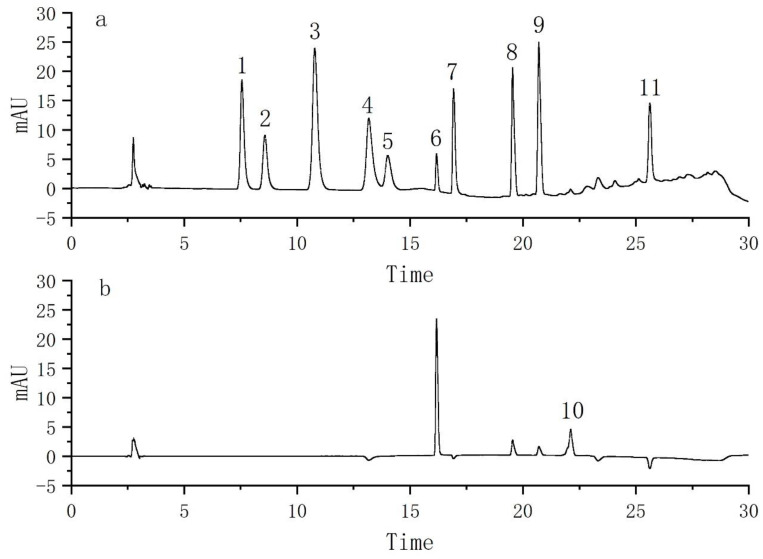
Chromatographic separation diagram of 11 food additives. The wavelength of 240 nm (**a**); The wavelength of 410 nm (**b**). (1) Acesulfame; (2) benzoic acid; (3) sorbic acid; (4) dehydroacetic acid; (5) saccharin sodium; (6) tartrazine; (7) amaranth; (8) sunset yellow; (9) Allura red; (10) brilliant blue; (11) erythrosine.

Finally, the optimum conditions were determined as follows: 40 mg C18−PS−DVB−Fe_3_O_4_ was used as an adsorbent to enrich 11 kinds of food additives by 100 times, then 20 mmol/L ammonium acetate solution was used as an eluent to elute the nanoparticles adsorbed with food additives, and then these were analyzed by HPLC. The obtained chromatogram is shown in Figure 12.

### 2.3. Analytical Performance of the Method

Under the magnetic solid phase extraction procedure, 11 kinds of food additives were analyzed by HPLC. It can be seen from Table 2 that 11 kinds of food additives were well enriched in the pretreatment stage. The linear range of 11 kinds of food additives was 0.1–10 μg/mL. The linear correlation coefficients were all above 0.9990. LODs and LOQs were 0.6–3.1 μg/L and 2.0–10.0 μg/L, respectively. The RSD was evaluated by measuring the solution in three groups of parallel experiments. The intra-day and inter-day RSD values of 11 food additives were less than 5.9% and 8.8%, respectively.

### 2.4. Regeneration and Reproducibility of C18−PS−DVB−Fe_3_O_4_

Using the same batch of C18−PS−DVB−Fe_3_O_4_ particles, the regeneration and stability of C18−PS−DVB−Fe_3_O_4_ particles as Mag-dSPE adsorbents were studied through six adsorption–desorption experiments. Before adsorption, the adsorbed particles will be washed with a desorption agent three times to remove the residual desorption agent in the last desorption process. As shown in Figure S3, C18−PS−DVB−Fe_3_O_4_ can be reused up to five times and the extraction efficiency will not be significantly reduced, but, in the sixth adsorption process, the adsorption performance will be greatly reduced. In addition, the preparation reproducibility of C18−PS−DVB−Fe_3_O_4_ was evaluated, and the results showed that the RSD of the three batches of adsorbents for 11 food additives were all less than 8.8%. Therefore, C18−PS−DVB−Fe_3_O_4_ had good regeneration performance and stability, and its preparation process also had good repeatability.

Furthermore, the preparative reproducibility of C18−PS−DVB−Fe_3_O_4_ was evaluated. The results show that the relative deviations of the recoveries for 11 food additives by using six batches of the sorbents are in the range of 3.7–9.4%. This indicates that the preparation procedure of C18−PS−DVB−Fe_3_O_4_ has good repeatability and reproducibility.

### 2.5. Method Validation and Real Sample Analysis

To explore the practicability of the established method, it was applied to detect 11 food additives in environmental water samples. Under optimal conditions, standard solutions of food additives synthesized in 11 were added to all samples to evaluate the matrix effects. Four groups of experiments were set up; the first group was the original water sample, the standard solution addition in the second group was 1.6 μg/L, the standard solution addition in the third group was 16 μg/L, and the standard volume addition in the fourth group was 80 μg/L. The obtained results are shown in Table 3. The recoveries were 86.53–106.32%, the inter-day RSD was 0.8–8.8%, and the intra-day RSD was 0.4–5.9%. Furthermore, Benzoic acid, temptation red, and erythrin were positive in the river water samples. The detected concentrations were 4.1 μg/L, 1.2 μg/L, and 7.0 μg/L, respectively. Therefore, the detection of food additives in environmental water samples is very necessary.

### 2.6. Compared with Other Methods

The performance of an extraction method based on C18−PS−DVB−Fe_3_O_4_ was compared with previously reported extraction methods for the determination of food additives. As can be seen in Table 4, the proposed method was demonstrated to have lower LODs values compared to other methods, and also had good RSD values and recovery.

In addition, this study also compared it with PS−DVB−Fe_3_O_4_ without C18 modification. The results showed that the materials without C18 modification were inferior to those without C18 modification in terms of detection limit and recovery. In the process of adsorption, PS−DVB−Fe_3_O_4_ belongs to physical adsorption and had no selectivity, while C18−PS−DVB−Fe_3_O_4_ belongs to chemical adsorption and had a certain selectivity, so the adsorbed material was purer, thus reducing the matrix effect and increasing the detection accuracy of the instrument.

## 3. Experimental

### 3.1. Reagents and Materials

Ferric chloride hexahydrate (FeCl_3_·6H_2_O), sodium acetate (NaAc), oleic acid (OA), ethylene glycol (EG), polyvinyl pyrrolidone (PVP), styrene (ST), divinylbenzene (DVB), 2,2’-Azobis(2-methylpropionitrile) (AIBN), sodium dodecyl sulfate (SDS), Dibutyl phthalate (DBP), polyvinyl alcohol (PVA), benzoyl peroxide (BPO), N, N-dimethyloctadecylamine, glycidyl methacrylate (GMA), and ammonium acetate of analytical grade were purchased from Sinopharm Chemical Reagent Co., Ltd. (Shanghai, China). Methanol of HPLC grade and the standard solution of acesulfame, amaranth, benzoic acid, tartrazine, saccharin sodium, sorbic acid, dehydroacetic acid, sunset yellow, allura red, brilliant blue, and erythrosine with concentrations of 1000 mg/L were purchased from Aladdin Bio-Chem Technology Co., Ltd. (Shanghai, China).

### 3.2. Equipment

The magnetic and morphological characteristics of C18−PS−DVB−Fe_3_O_4_ were carried out by using a Lake Shore 7404 vibrating sample magnetometer (VSM) (Westerville, OH, USA) and Zeiss Supra 55 Field emission scanning electron micrographs (SEM) (Zeiss, Oberkochen, Germany). X-ray photoelectron spectroscopy (XPS) data were obtained by Thermo Escalab 250XI (Thermo Escalab, Waltham, MA, USA). The specific surface area and pore size of the adsorbent were measured and calculated by Brunner–Emmet–Teller (BET) and Barrett–Joyner–Halenda (BJH) methods (Micromeritics, Norcross, GA, USA). Furthermore, C18−PS−DVB−Fe_3_O_4_ was also analyzed by Fourier transform infrared spectroscopy (FTIR).

### 3.3. Synthesis of PS−DVB−Fe_3_O_4_

#### 3.3.1. Synthesis of Fe_3_O_4_

The Fe_3_O_4_ nanoparticles were prepared by the solvothermal method [47]. Briefly, 2.0 g FeCl_3_·6H_2_O and 6.0 g NaAc were dissolved in 40 mL EG under ultrasonication, and then it was transferred to a Teflon-lined stainless steel autoclave (100 mL). Afterwards, the autoclave was heated to 200 °C and was maintained for 6 h, then cooled naturally to room temperature. Under the action of a magnetic field, the obtained Fe_3_O_4_ particles were washed with deionized water and anhydrous ethanol and were then dried in a vacuum oven at 60 °C for 12 h.

#### 3.3.2. OA Coated Fe_3_O_4_ Nanoparticles

Furthermore, 1.0 g of Fe_3_O_4_ as prepared above was dispersed in 200 mL of anhydrous ethanol. Then, 5.0 mL OA was slowly dripped at a reaction temperature of 80 °C and a stirring speed of 500 rpm. And then, the reaction lasted for 1.0 h. At the end of the reaction, under the action of a magnetic field, the obtained particles were washed with deionized water and anhydrous ethanol and then vacuum dried at 60 °C for 12 h. Save the brown particles and set them aside.

#### 3.3.3. Synthesis of PS−DVB−Fe_3_O_4_

Then, the 1.0 g Fe_3_O_4_ nanoparticles coated with oleic acid, 1.0 g PVP, 100 mL 95% ethanol-water, 6.0 g ST, and 0.8 g AIBN were uniformly mixed and transferred to a 250 mL three-neck flask. The stirring speed was 150 rpm. The reaction was carried out with nitrogen for 24 h. Then, the obtained particles were washed with deionized water and anhydrous ethanol ethylene several times under the action of a magnetic field.

Afterwards, the particles were transferred to the three-neck flask of 500 mL and added to the 20 mL SDS (0.2%, *m*/*v*) solution. DBP was added to 30 mL SDS (0.2%, *m*/*v*) solution and emulsified for 1 h and then poured into the three-neck flask at a stirring speed of 150 rpm at 30 °C for 24 h. Additionally, 2.5 g PVA, 0.8 g SDS, 0.2 g BPO, 5.0 mL GMA, 7.5 mL DVB, and 14 mL toluene were added to 250 mL deionized water, emulsified for 2 h, poured into the three-neck flask, and stirred at 30 °C. The reaction was carried out with nitrogen at 70 °C for 24 h. The obtained particles were washed several times with hot water and anhydrous ethanol under the action of a magnetic field.

#### 3.3.4. Synthesis of C18−PS−DVB−Fe_3_O_4_

Furthermore, 25 mL methanol was used as a solvent, adding 1.0 g of PS−DVB−Fe_3_O_4_ polymer composite material, and then undergoing ultrasonic dispersion for 5.0 min. Under the condition of 80 °C, condensation reflux, and 2.5 mL N, N-dimethyloctadecylamine was added to the reaction system under the stirring speed of 500 rpm for 8 h. After the reaction was completed, the C18−PS−DVB−Fe_3_O_4_ particles were washed with deionized water and then washed with anhydrous ethanol several times under the action of a magnetic field. Then, it was dried in a vacuum drying box at 60 °C for 12 h. Finally, the C18−PS−DVB−Fe_3_O_4_ nanoparticles were obtained.

### 3.4. HPLC-DAD Analysis

The chromatographic separation was performed on a ChromCore^TM^AQC18 (5 μm, 4.6 × 250 nm) by using 20 mmol/L ammonium acetate in water as eluent (A) and methanol as eluent (B) as the mobile phase. The detector wavelengths were 240 nm and 410 nm, and the temperature of the column thermostat was 20 °C. The injection volume was 10 μL. The separation was accomplished at a constant flow of 1.0 mL/min. The linear gradient conditions were shown in Table S1.

### 3.5. Method Validation

#### 3.5.1. Standard Preparation

Stock standard solution at the concentration of 100 mg/L for acesulfame, amaranth, benzoic acid, tartrazine, saccharin sodium, sorbic acid, dehydroacetic acid, sunset yellow, allura red, brilliant blue, and erythrosine were prepared by diluting the standard solution (1000 mg/L) in ultra-pure water. Five groups of mixed standard solutions with the concentration of 0.1 μg/mL, 0.5 μg/mL, 1.0 μg/mL, 5.0 μg/mL, and 10 μg/mL were prepared by diluting the standard solution (100 mg/L) with ultra-pure water, respectively.

#### 3.5.2. Mag-dSPE Procedure

Firstly, the environmental water sample was filtered with a 0.45 μm filter membrane to remove the particles in the environmental sample. Then, 40 mg C18−PS−DVB−Fe_3_O_4_ was dispersed in 100 mL environmental sample solution and stirred for 20 min to make the analyte fully adsorbed by magnetic nanoparticles. Then, under the action of a magnetic field, the solid–liquid separation was completed. The separated particles were eluted with 20 mmol/L ammonium acetate–methanol solution (1.0 mL). The collected eluent was passed through a 0.45 μm filter membrane and transferred to the injection bottle. The final solution was injected into the HPLC system for progressive analysis.

#### 3.5.3. Validation Parameters

In this experiment, the linearity, precision, reproducibility, and accuracy of the method were evaluated. Through the analysis of the standard samples of five concentrations, the standard curves of the peak area and concentration were obtained. The obtained standard curve was used to correct the spiked samples. When the signal-to-noise ratio (S/N) was 3 and 10, respectively, the LOQs and LODs values were estimated according to the baseline noise method. The accuracy of the method was expressed by recovery, and the precision was expressed by intra-day and inter-day RSD. The inter-day RSD was obtained by repeating experiments for 6 times in 6 days. The intra-day RSD was obtained by repeating experiments for 6 times in one day.

## 4. Conclusions

In summary, a novel magnetic nanomaterial of C18−PS−DVB−Fe_3_O_4_ was prepared. And C18−PS−DVB−Fe_3_O_4_ was used as an adsorbent of Mag-dSPE and then combined with high-performance liquid chromatography. This method was used to detect 11 food additives in environmental water samples. Under the optimum conditions, the recovery rate of 11 food additives in environmental water samples was between 86.53 and 106.32%, and the LODs were below 3.1 μg/L. This study shows that this material can effectively enrich trace food additives from environmental water samples and has potential application value. However, C18−PS−DVB−Fe_3_O_4_ still needs improvement in some areas, such as the particle uniformity not being good enough. After that, the research direction can adjust the particle size of C18−PS−DVB−Fe_3_O_4_ and prepare uniform nanoparticles. Then, it will be used as a substitute for silicon-based materials for the filling of chromatographic columns.

## Figures and Tables

**Table 1:** Nitrogen analysis of C18−PS−DVB−Fe_3_O_4_.

Material	Gross Mass (g)	N%	O%	Epoxy Group Content (mmol)	N, N-Dimethyloctadecylamine
PS−DVB−Fe_3_O_4_	1.0	/	12.23	2.546	/
C18−PS−DVB−Fe_3_O_4_	1.0	1.45	8.46	1.766	1.035

**Table 2:** Linear Equation, LODs, and RSD of 11 food additives (*n* = 6).

Target	Linear Range (μg/mL)	Linear Equation	R^2^	LOD (μg/L)	LOQ (μg/L)
Acesulfame	0.1–10	Y = 22.86X + 7.068	0.9991	2.3	7.6
Benzoic Acid	0.1–10	Y = 15.31X + 3.012	0.9994	2.0	6.6
Sorbic Acid	0.1–10	Y = 20.27X + 5.462	0.9998	1.8	5.9
Dehydroacetic Acid	0.1–10	Y = 6.585X + 8.065	0.9992	2.7	8.9
Saccharin Sodium	0.1–10	Y = 7.042X + 1.530	0.9992	2.9	9.6
Tartrazine	0.1–10	Y = 2.327X + 0.5170	0.9997	3.0	9.9
Amaranth	0.1–10	Y = 15.720X + 1.708	0.9995	2.3	7.6
Sunset Yellow	0.1–10	Y = 19.166X + 3.887	0.9995	1.2	4.0
Allura Red	0.1–10	Y = 24.529X + 5.627	0.9993	0.6	2.0
Brilliant Blue	0.1–10	Y = 18.27X + 1.075	0.9991	3.1	10.0
Erythrosine	0.1–10	Y = 13.25X − 0.2473	0.9996	2.4	7.9

**Table 3:** Content, recovery rate, and relative standard deviation of 11 food additives in environmental water samples (*n* = 6).

Analytes	Sample Concentration (μg/L)	Added (μg/L)	Recovery %	RSD %	
				Inter-Day	Intra-Day
Acesulfame	—	1.6	97.60	1.5	2.3
		16.0	96.45	3.8	5.8
		80.0	99.21	2.9	0.4
Benzoic acid	4.1	1.6	98.02	5.8	3.8
		16.0	101.53	0.8	4.2
		80.0	99.63	6.5	5.5
Sorbic acid	—	1.6	98.25	2.9	1.9
		16.0	95.65	3.2	3.6
		80.0	96.02	1.8	1.1
Dehydroacetic acid	—	1.6	102.31	2.9	1.6
		1.6.0	106.32	3.7	4.2
		80.0	105.27	4.1	5.9
Saccharin Sodium	—	1.6	98.24	5.8	1.3
		16.0	97.35	1.9	5.1
		80.0	100.62	4.1	4.8
Tartrazine	—	1.6	99.32	3.2	3.7
		16.0	101.52	6.2	1.2
		80.0	99.61	4.7	1.4
Amaranth	—	1.6	99.45	2.3	3.7
		16.0	102.90	1.5	2.2
		80.0	104.80	6.9	4.1
Sunset Yellow	—	1.6	91.40	8.8	2.7
		16.0	90.55	4.7	5.6
		80.0	93.93	3.6	0.5
Allura Red	1.2	1.6	93.90	1.1	5.5
		16.0	94.77	3.9	3.8
		80.0	95.63	2.3	1.2
Brilliant Blue	—	1.6	95.10	3.8	0.8
		16.0	88.23	4.1	0.7
		80.0	90.52	2.6	4.2
Erythrosine	7.0	1.6	89.71	1.3	4.8
		16.0	86.53	1.7	0.9
		80.0	87.84	7.5	3.4

**Table 4:** Comparison of the proposed method with other previously reported methods for the determination of food additives in different samples.

Sample	Sorbent	Detection Method	Recovery (%)	RSD (%)	LOD (μg/L)	Ref.
The aqueous food	Silica C18 cartridge	LC-DAD	78–104%	2.0–7.7	34,000–71,000	[41]
Food packages	Silica C18 cartridge	HPLC-UV	67.48–108.5%	2.7–9.8	900–1720	[42]
Plastic packaging	Fe_3_O_4_@CTAB	HPLC-UV	84.5–98.9%	0.4–2.4	90–170	[43]
Soft drink samples	Al_2_O_3_	HPLC-DAD	90.4–109.2	0.7–5.1	9.5–9.9	[44]
Drink samples	Fe_3_O_4_@rGO	HPLC	88.9–95.9	<2.7	10.0–15.9	[45]
Liquor samples	Decanoic acid coated-Fe_3_O_4_ NPs	HPLC	88.9–105.4	2.9–3.8	0.9–2.4	[46]
River water	PS−DVB−Fe_3_O_4_	HPLC-DAD	43.6–67.3	2.5–9.8	15.1–26.7	In this work
River water	C18−PS−DVB−Fe_3_O_4_	HPLC-DAD	86.5–106.3	0.4–8.8	0.6–3.1	In this work

## Data Availability

Details are available from authors.

## Supplementary Materials

The following supporting information can be downloaded at https://www.mdpi.com/article/10.3390/molecules29153675/s1, Figure S1: Nitrogen adsorption–desorption isotherms of C18−PS−DVB−Fe_3_O_4_ (a), The corresponding pore size distribution curves of C18−PS−DVB−Fe_3_O_4_ (b). Figure S2: Nitrogen adsorption–desorption isotherms of PS−DVB−Fe_3_O_4_. Figure S3: Reusability of C18−PS−DVB−Fe_3_O_4_ for extraction of synthetic food additives (*n* = 6), Table S1: Mobile phase gradient elution condition.
