# Supplementary material for: Fast Determination of Eleven Food Additives in River Water Using C18 Functionalized Magnetic Organic Polymer Nanocomposite Followed by High-Performance Liquid Chromatography
Source: Molecules. 2024 Aug 2;29(15):3675. doi: 10.3390/molecules29153675 (PMC11314223; doi:10.3390/molecules29153675)
Supplement: Supplementary file 1 [file molecules-29-03675-s001.zip › molecules-3107596-supplementary.pdf]

Supporting information

Figure S1. Nitrogen adsorption-desorption isotherms of the C18-PS-DVB-Fe_3O_4 (a). The corresponding pore size distribution curves of the C18-PS-DVB-Fe_3O_4 (b).

Figure S2. Nitrogen adsorption-desorption isotherms of the PS-DVB-Fe_3O_4

Figure S3. Reusability of C18-PS-DVB-Fe_3O_4 for extraction of synthetic food additives. (n=6)

Table S1 Mobile phase gradient elution condition.

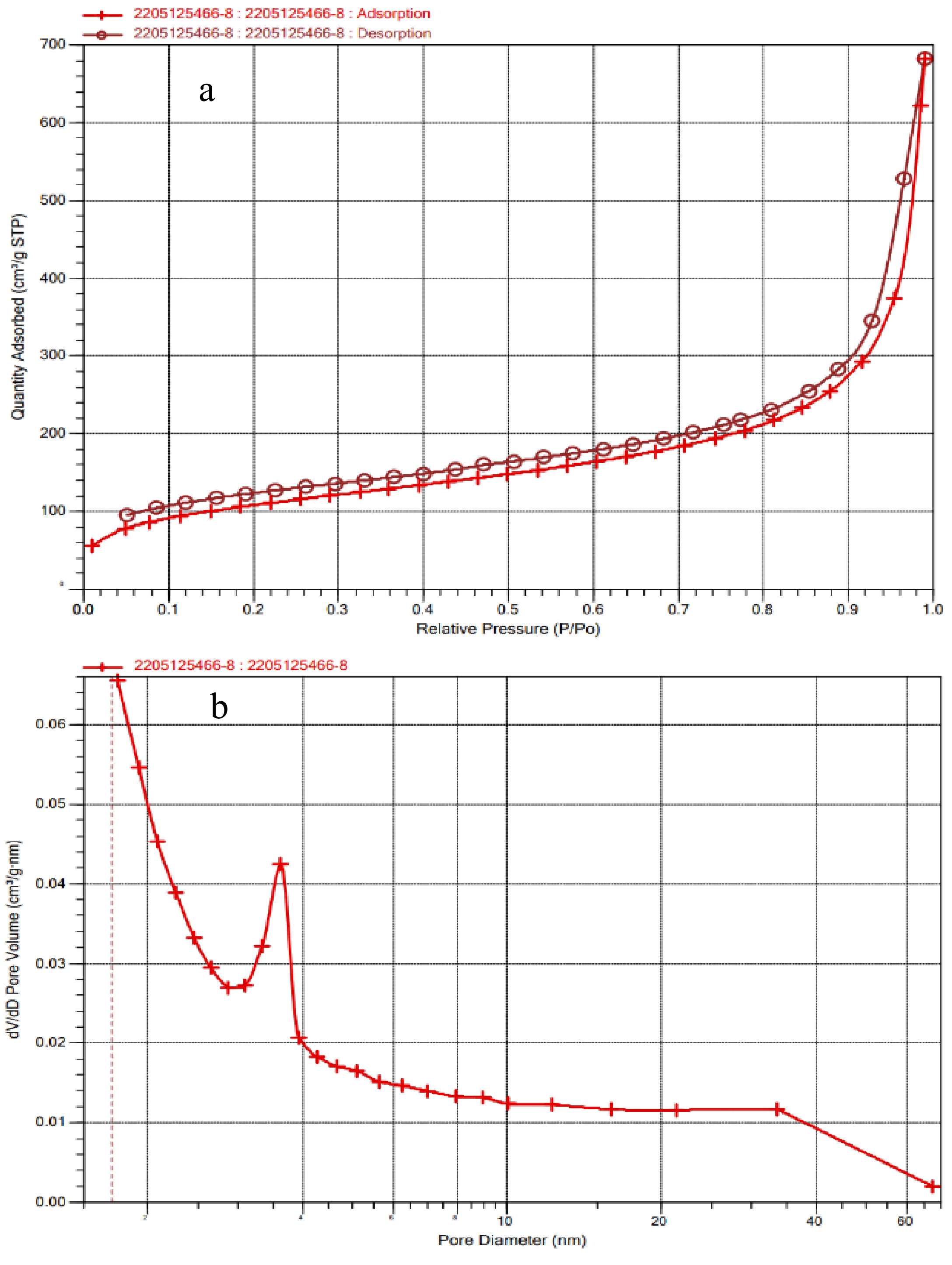

Figure S1. Nitrogen adsorption-desorption isotherms of the C18-PS-DVB-Fe_3O_4 (a). The corresponding pore size distribution curves of the C18-PS-DVB-Fe_3O_4 (b).

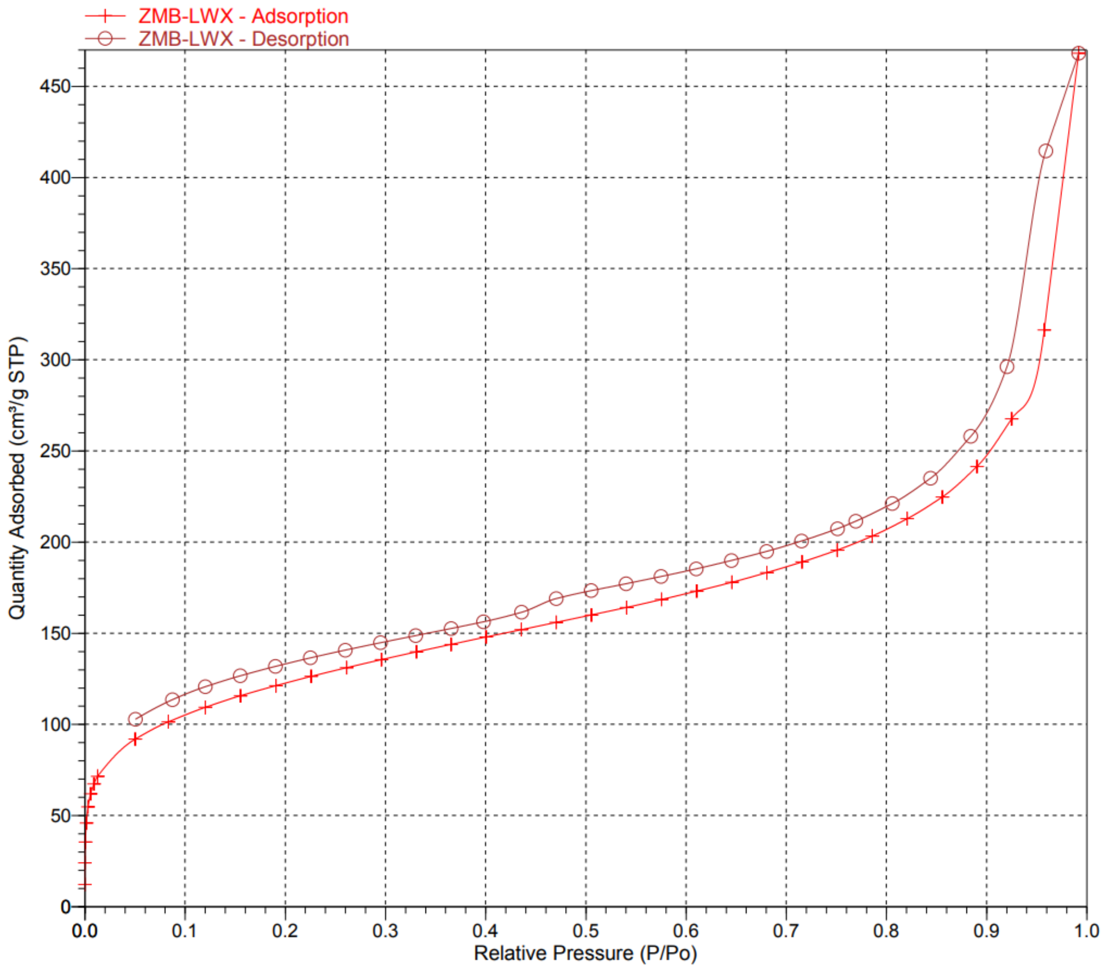

Figure S2. Nitrogen adsorption-desorption isotherms of the PS-DVB-Fe_3O_4

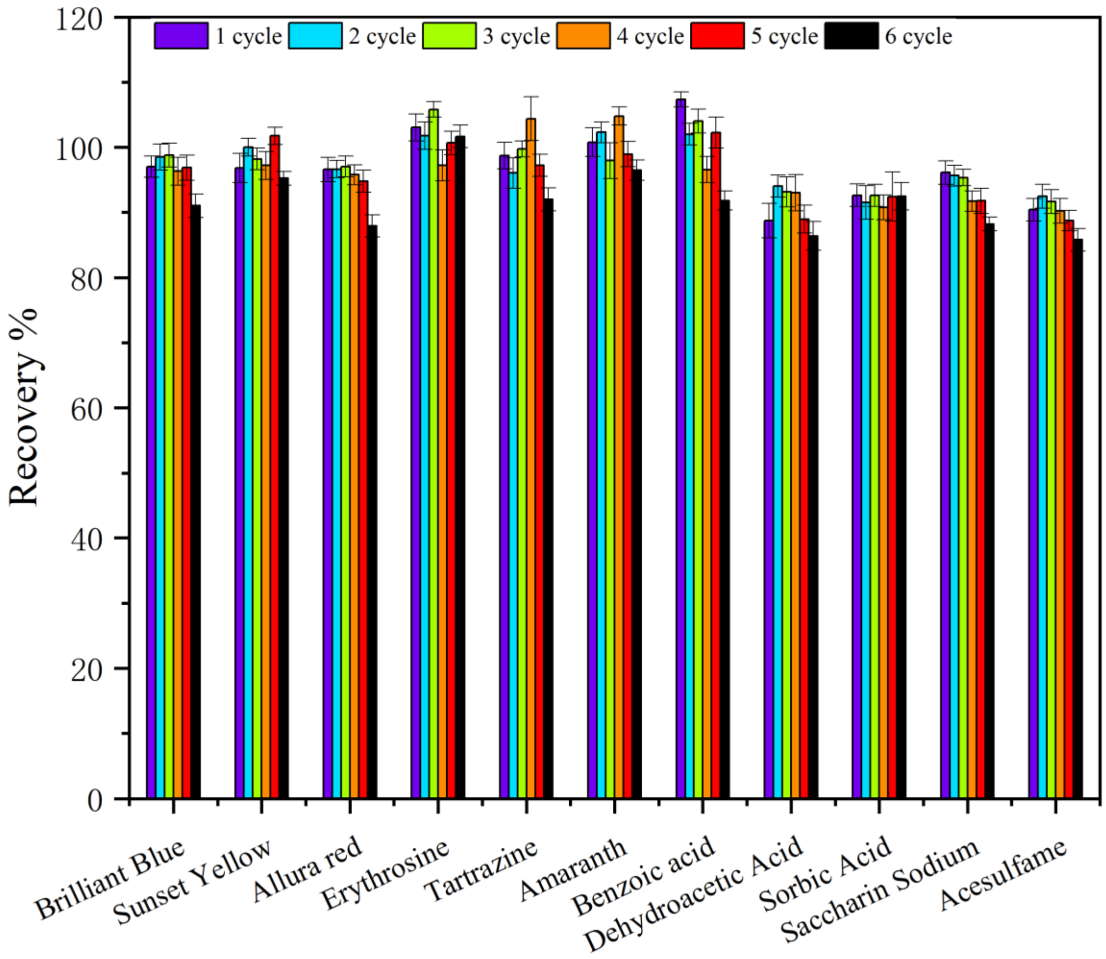

Figure S3. Reusability of C18-PS-DVB-Fe_3O_4 for extraction of synthetic food additives. (n=6)

Table S1 Mobile phase gradient elution condition.

Time/min	20 mmol/L ammonium acetate in water (A)	Methanol (B)
0	95%	5%
10	92%	8%
25	5%	95%
30	95%	5%
35	95%	5%
